# High Tumoral CD24 Expression and Low CD3^+^ Tumor-Infiltrating Lymphocytes as a Biomarker for High-Risk Locally Advanced Nasopharyngeal Carcinoma

**DOI:** 10.3390/cancers17132094

**Published:** 2025-06-23

**Authors:** Hazem Ghebeh, Jumanah Y. Mirza, Shamayel F. Mohammed, Hatim A. Khoja, Mohammad Anas Dababo, Muruj E. Tukruni, Muhammad S. Anwar, Mohammed Aldehaim, Nasser Al-Rajhi

**Affiliations:** 1 Cell Therapy and Immunobiology Department, King Faisal Specialist Hospital and Research Centre, Riyadh 11211, Saudi Arabia; jmirza@kfshrc.edu.sa (J.Y.M.); n3520664@kfshrc.edu.sa (M.E.T.); 2 College of Medicine, Al-Faisal University, Riyadh 115332, Saudi Arabia; 3 Department of Pathology, King Faisal Specialist Hospital and Research Centre, Riyadh 11211, Saudi Arabia; smohammed@kfshrc.edu.sa (S.F.M.); hkhoja@kfshrc.edu.sa (H.A.K.); mdababo@kfshrc.edu.sa (M.A.D.); 4 Department of Medical Oncology, King Faisal Specialist Hospital and Research Centre, Riyadh 11211, Saudi Arabia; manwar2@kfshrc.edu.sa; 5 Department of Radiation Oncology, King Faisal Specialist Hospital and Research Centre, Riyadh 11211, Saudi Arabia; maldehaim@kfshrc.edu.sa (M.A.); nrajhi@kfshrc.edu.sa (N.A.-R.)

**Keywords:** nasopharyngeal carcinoma, tumor-infiltrating lymphocytes, CD3

## Abstract

**Introduction:** The limited value of TNM staging in locally advanced (LA) nasopharyngeal carcinoma (NPC), defined as stage III or IV NPC, highlights the need for prognostic markers. Here, we aimed to evaluate the ability of the cancer stem cell markers, BMI1, ALDH1, CD44, and CD24, and the epithelial–mesenchymal transition marker, vimentin, as novel prognostic markers in LA-NPC. **Methods:** A cohort of 83 patients with LA-NPC, previously recruited for another trial with a different goal, was used. The marker expression in tissue sections was evaluated using immunohistochemistry, and the association of expression with survival outcomes was analyzed using the Kaplan–Meier method and Cox regression analysis. **Results:** The expression of BMI1 and ALDH1 was not associated with survival. The tumoral expression of CD24, CD44, and vimentin was significantly associated with disease-free survival (DFS), whereas only the expression of CD24 was also significantly associated with overall survival (OS). Indeed, CD24 expression emerged as an independent prognostic factor as it was associated with survival in univariate and multivariate Cox regression analysis. Interestingly, combining the status of CD24 expression in tumor cells with the density of CD3^+^ tumor-infiltrating lymphocytes (TIL), a microenvironment/immune-related marker, identified a high-risk subgroup with the worst DFS and OS. **Conclusions:** These results suggest the utility of combining tumoral CD24 expression and the CD3^+^ TIL density as a prognostic factor in LA-NPC, with potential application in disease management and future trial design. Our findings also highlight the potential of combining immune- and cancer stem cell-related factors in personalized treatment strategies for cancer.

## Simple Summary

While the TNM staging system is widely used in solid tumors, it has limitations in locally advanced nasopharyngeal carcinoma (LA-NPC), highlighting the need for markers to identify high-risk LA-NPC patients. In this study, we evaluated the ability of certain oncogenic-trait markers to identify high-risk patients using a cohort of 83 LA-NPC patients. The results demonstrated that the tumoral expression of CD24 was significantly associated with survival. Interestingly, by combining the status of CD24 expression in tumor cells with the density of T-lymphocytes infiltrating the tumor—an immune-related marker—we identified a high-risk subgroup with the worst survival outcomes. These findings suggest the utility of combining tumoral CD24 expression with T-lymphocyte density as a combined marker for LA-NPC, which has potential applications in disease management and future trial design.

## 1. Introduction

Nasopharyngeal carcinoma (NPC), a cancer that arises from the nasopharynx, is distinct from other types of head and neck cancers due to the role of the Epstein–Barr virus (EBV) in the disease biology and the striking geographical differences in its incidence [1]. NPC is more common in Southeast China, parts of the Middle East, and North Africa, where most patients present with locally advanced disease. Importantly, locally advanced nasopharyngeal carcinoma (LA-NPC), defined as stage III or IV, is the most common form of disease in Saudi Arabia. While a large percentage of patients with LA-NPC are successfully managed using concurrent chemoradiotherapy (CCRT), relapse, metastatic disease, and death occur in a subset of patients. Prognostic markers that can identify the patients with increased relapse risk are lacking, especially in the context of the limited use of the traditional TNM staging commonly used in cancers. In a previous study investigating the ability of several immune-related parameters as potential prognostic markers, we reported that the status of low CD3^+^ tumor-infiltrating lymphocytes (TIL) in the tumor bed was highly prognostic of relapse and worse survival [2].

Cancer stem cells (CSCs) define a subset of tumor cells with self-renewal ability that can reform the tumor with all its heterogeneity following primary tumor implantation into a new host [3]. In addition to their role in tumor maintenance, CSCs are therapy-resistant and exhibit high metastatic capability [4]. In NPC, multiple CSC markers have been described, including BMI1 [5], ALDH1 [6], CD44 [7], CD24 [8], and CD44/CD24 [8,9]. Furthermore, ALDH1/CD44 have been used to describe CSCs in other head and neck cancers [10]. However, the prognostic ability of these CSC markers has not been established in LA-NPC.

In the present study, we tested the expression of several CSC markers side-by-side and evaluated the association of each marker with the survival of patients with LA-NPC. Among the tested markers, CD24 positivity in ≥30% of the tumor cells was significantly associated with disease-free survival (DFS), metastasis-free survival (MFS), and overall survival (OS). Interestingly, when the CD24 positivity was used together with another immune prognostic marker (CD3^+^ TIL status), a subgroup of NPC tumors that were positive for CD24 expression and had low CD3^+^ TIL in the tumor microenvironment had the worst DFS, MFS and OS. We propose that this combination biomarker can be used to guide the management of patients with LA-NPC and highlight the need for future clinical trials to evaluate the efficacy of more aggressive or novel treatment regimens in this group of high-risk NPC patients.

## 2. Methods

### 2.1. Study Design and Patient Selection

This was a retrospective, single-institution cohort study evaluating the efficacy of specific CSC markers in predicting survival in a cohort of patients with LA-NPC who participated in a previous randomized controlled trial with a different goal [11]. The previous randomized phase II–III trial aimed to evaluate the efficacy of low-dose fractionated radiation with induction neoadjuvant chemotherapy followed by CCRT in patients with LA-NPC [11]. As previously reported, the survival rate was not significantly different between the two groups in the previous study, and therefore, the tissue was utilized for this current study [11]. Tissue blocks were available for analysis for 83 of the 108 patients enrolled in the previous study. Even with the available tissue blocks, tissues were completely exhausted before evaluating the expression of some markers (n = 1 for BMI1 and n = 9 for ALDH1, CD24, and CD44).

### 2.2. Immunohistochemistry

Formalin-fixed paraffin-embedded NPC tissue blocks obtained at the time of diagnosis were used for immunohistochemistry. Briefly, 4 µm thick tissue sections were deparaffinized, rehydrated, and immunohistochemically stained for CD3 and vimentin using a fully automated Ventana Benchmark Ultra system (Ventana/Roche), as previously described [2], or immunohistochemically stained for BMI1, CD24, ALDH1 CD44 using a manual method, as described below.

For BMI1 staining, a primary anti-mouse BMI1 antibody (dilution, 1:500; clone F6; Merck) was used with the Envision^®^ anti-mouse secondary antibody (Agilent, Santa Clara, CA, USA). For double immunostaining for CD24/CD44 and ALDH1/CD44, the sections were blocked for biotin/avidin, followed by protein block using 10% goat serum. Next, the sections were incubated overnight at 4 °C with the following primary antibodies: mouse anti-CD24 (clone ML5) or mouse anti-ALDH1 (dilution, 1:200; clone 44/ALDH; BD Biosciences) mixed with rabbit anti-CD44 antibody (dilution, 1:500; catalog no, HPA005785; Sigma, St. Louis, MO, USA). The sections were then incubated with the 4plus goat anti-mouse IgG secondary antibody (catalog no, GM601H), followed by incubation with the 4plus streptavidin alkaline phosphatase (catalog no, AP605H), both from Biocare Medical (Pacheco, CA, USA). Next, the sections were incubated with the Envision^®^ anti-rabbit secondary antibody (Agilent), and the signals were visualized using sequential staining with Fast Red followed by diaminobenzidine (both from Biocare Medical).

### 2.3. Verification of Antibodies Used in Manual Immunohistochemistry

The specificity of antibodies used in manual immunohistochemistry was verified in sections of formalin-fixed paraffin-embedded cell lines, including U-937 leukemia and MDA-MB-231 breast cancer cells as high BMI1 expression controls [12] and MDA-MB-468 breast cancer cells as low BMI1 expression control [13] (Supplementary Figure S1). A549 lung cancer cells were used as a positive control for ALDH1 and CD44 [14], while MCF-7 breast cancer cells were a negative control for ALDH1 and CD44. SK-BR-3 breast cancer cells were used as a positive control for CD24, while MDA-MB-231 cells were a negative control for CD24 (also negative for ALDH1) and a positive control for CD44 [15,16]. Finally, HCC1937 breast cancer cells were used as a control for double positivity of CD44 and CD24.

### 2.4. Pathologic Scoring

Scoring of CD3^+^ TIL density was performed as previously described [2,17]. BMI1 intensity was scored as follows: 0, negative; 1, mild; 2, moderate; 3, intense staining intensities. BMI1 staining intensity was further dichotomized into negative for scores 0–1 and positive for scores 2–3. The sections stained for ALDH1, CD44, and CD24 were scored using 5–10% increments, with 10%, 70%, and 30% as cutoffs for ALDH1, CD44, and CD24, respectively. Furthermore, H-scores were used in additional analyses for ALDH1 and CD24 staining, as previously reported [10], with an H-score of 90 used as the cutoff for both markers. Similarly, the sections coimmunostained for ALDH1/CD44 or CD24/CD44 were scored using 5–10% increments, with 10% as the cutoff for both combinations. All cutoffs were determined using the receiver operating characteristic (ROC) curve/Youden index (Supplementary Figure S2). H-score used for CD24 immunostaining, for which the cutoff (x = 90) corresponded to the second-highest Youden index.

### 2.5. Statistical Analysis

The association of specific CSC markers with survival outcomes was evaluated using the Cox proportional hazards model. Survival plots were generated using the Kaplan–Meier method, and the curves were compared using the log-rank test. Time was censored for patients who were alive or disease-free at the last follow-up. Categorical variables were compared using Fisher’s exact test. All analyses, including the ROC curve/Youden index analyses, were performed using JMP statistical software (version 15; SAS Institute, Cary, NC, USA), and a *p*-value of 0.05 was set as the threshold for significance.

## 3. Results

### 3.1. Patient Characteristics

In the present study, the majority of the patients were males with large tumors (T3 and T4) and advanced lymph node involvement (N), consistent with LA-NPC (Supplementary Table S1). The tumors were predominantly nonkeratinizing undifferentiated carcinoma, i.e., World Health Organization (WHO) histological subtype III. In the overall cohort, the median follow-up duration after the initial diagnosis was 7.7 years; 23 patients (28%) relapsed, including 6 patients with local or locoregional relapse and 17 patients (20%) with systemic relapse; and 12 patients (14%) eventually died from the disease.

### 3.2. CD24 Expression Is Significantly Associated with Survival

**Figure 1:**
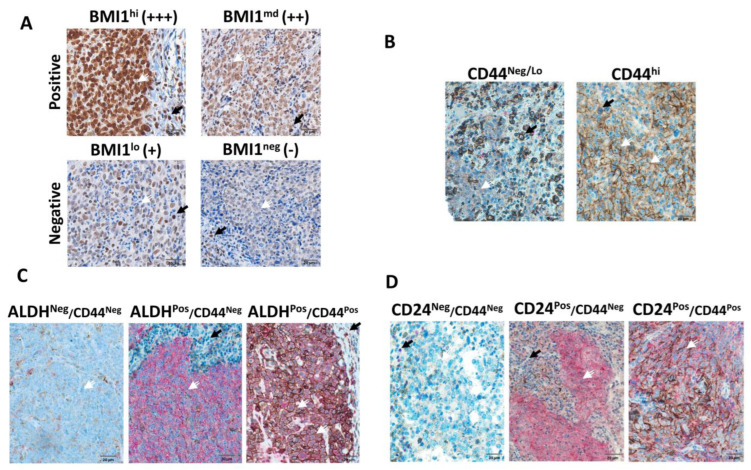
Representative images of NPC tumor sections evaluated for the expression of CSC markers using immunohistochemistry. Representative images of tumor tissue sections showing nuclear BMI1 (**A**), membranous CD44 (**B**), primarily cytoplasmic ALDH1, alone or in combination with CD44 (**C**), and primarily cytoplasmic CD24, alone or in combination with membranous CD44 (**D**) in NPC tissue sections. White arrows indicate tumor cells, whereas black arrows indicate immune infiltrating cells.

**Figure 2:**
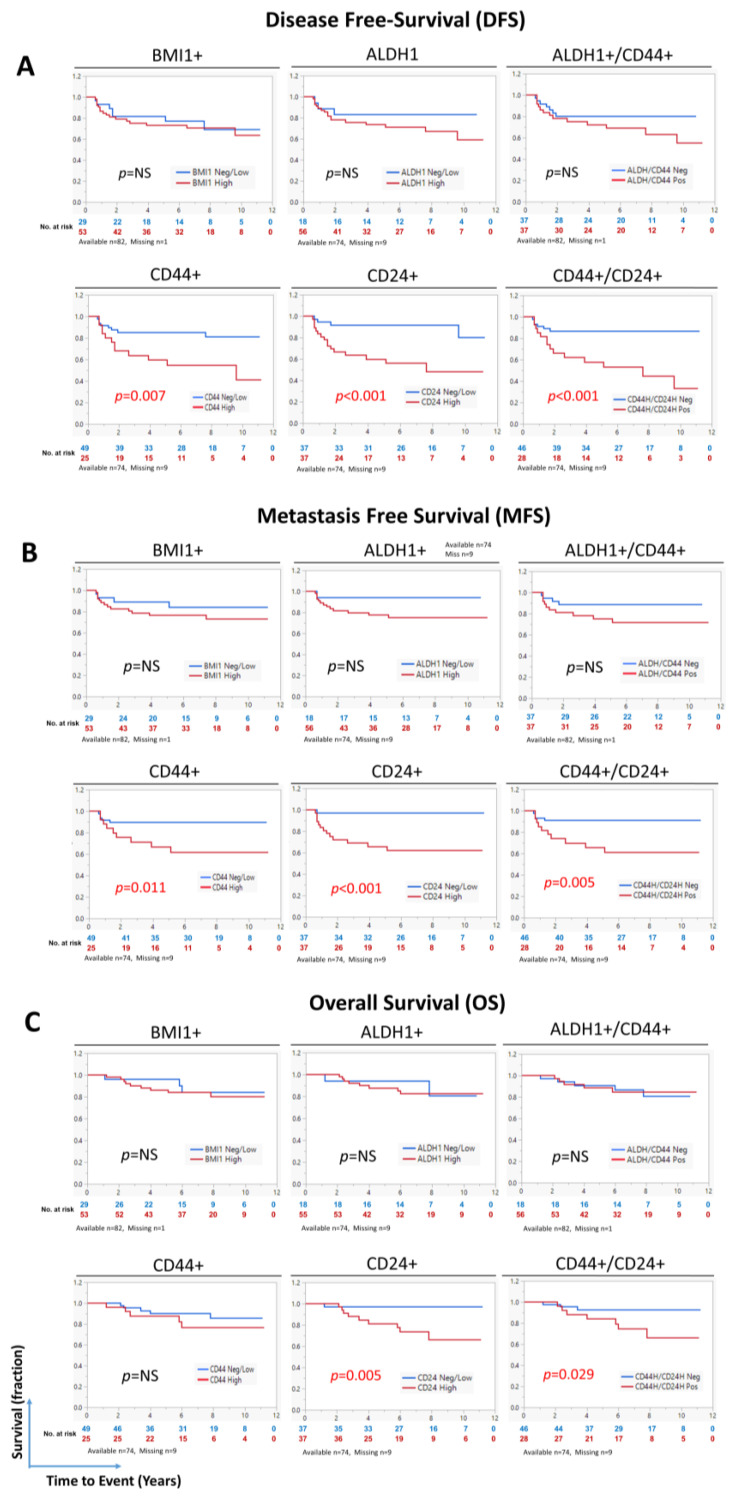
Prognostic ability of CSC markers in LA-NPC. Kaplan–Meier survival curves showing (**A**) disease-free survival (DFS), (**B**) metastasis-free survival (MFS), and (**C**) overall survival (OS) in patients with locally advanced nasopharyngeal carcinoma (LA-NPC) categorized according to the cancer stem cell (CSC) marker expression status. Statistical significance was evaluated using the log-rank test. The numbers below the curve represent the number of patients who are still at risk of experiencing the event (color coded to correspond to each group in the analysis).

In NPC tissue sections, the nuclear BMI1 intensity in tumor cells varied between low intensity (score of 0 or 1) to high intensity (score of 2 or 3) (white arrows, Figure 1). Conversely, the intensity of BMI1 in immune cells was relatively consistent and used as an internal control (black arrow). In 65% of the cases, the BMI1 expression was high in tumor cells and did not significantly correlate with DFS, MFS, or OS (Figure 2). Furthermore, the BMI1 expression did not significantly correlate with the expression of other CSC markers or other clinicopathologic characteristics (Supplementary Table S2).

Membranous CD44 was overexpressed in tumor cells in 34% of the cases. In addition, CD44 overexpression was observed in immune infiltrating cells. High CD44 expression in tumor cells significantly correlated with DFS (*p* = 0.007) and MFS (*p* = 0.011) but not with OS (Figure 2). A significant correlation existed between CD44 expression and older age (*p* < 0.001) as well as high T stage (*p* = 0.038) (Supplementary Table S3).

ALDH1 exhibited a predominantly cytoplasmic expression pattern and was overexpressed in tumor cells in 76% of the cases (Figure 1). Additionally, ALDH1 was expressed by some histiocytes/macrophages (Supplementary Figure S3). ALDH1 expression in tumor cells did not significantly correlate with patient survival (Figure 2). ALDH1 expression significantly correlated with CD24/CD44 coexpression (*p* = 0.049), which was not observed with other CSC markers or other clinicopathologic characteristics (Supplementary Tables S2 and S3). Given that the ALDH1 staining intensity was heterogeneous (Supplementary Figure S4A), we also scored ALDH1 expression using the H-score, as previously described [18]. As shown in Supplementary Figure S5, ALDH1 expression determined using the H-score did not correlate with DFS, MFS, or OS.

Tumor cells that were positive for both ALDH1 and CD44 existed in 50% of the cases studied in this cohort. However, the ALDH1/CD44 coexpression did not correlate with survival (Figure 2) or the expression of other CSC markers or clinicopathologic characteristics (Supplementary Table S2).

CD24, mostly cytoplasmic, was overexpressed in 50% of the cases, and CD24 expression significantly correlated with DFS (*p* < 0.001), MFS (*p* < 0.001), and OS (*p* = 0.005) (Figure 2) but not with the expression of other CSC markers or clinicopathologic characteristics (Supplementary Table S3). Given the heterogeneous CD24 staining intensity (Supplementary Figure S4B), we also scored the sections using the H-score. As shown in Supplementary Figure S4, CD24 expression determined using the H-score significantly correlated with DFS (*p* < 0.001), MFS (*p* = 0.002), and OS (*p* = 0.001) (Supplementary Figure S5).

In 38% of the cases, the tumor cells coexpressed CD24 and CD44 (Figure 1D). The CD24/CD44 coexpression significantly correlated with DFS (*p* < 0.001), MFS (*p* = 0.005), and OS (*p* = 0.029) (Figure 2) and with ALDH1 expression (*p* = 0.049) (Supplementary Tables S2 and S3).

Altogether, the analyses of CSC markers revealed that high CD24 expression, using two scoring approaches, and whether CD24 alone or in combination with high CD44 expression, correlated significantly with shorter DFS, MFS, and OS.

### 3.3. Expression of Vimentin Is Associated with Shorter DFS

**Figure 3:**
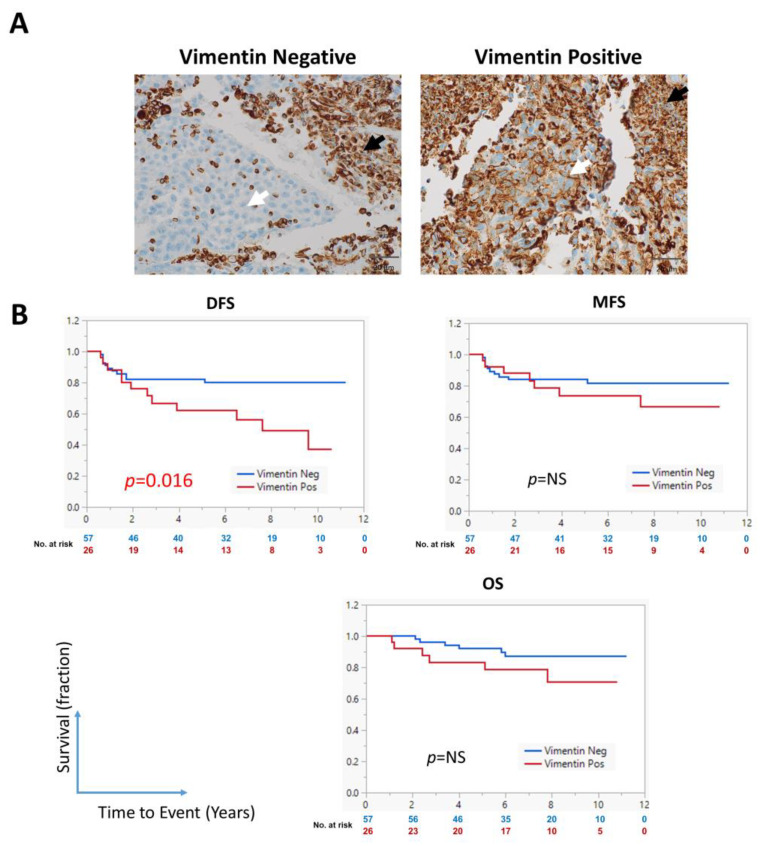
Association of vimentin expression with prognosis in patients with LA-NPC. (**A**) Representative images of LA-NPC tissue specimens with vimentin expressing (right) or not expressing (left) tumor cells (white arrows). Immune infiltrating cells are positive (black arrows). (**B**) Kaplan–Meier survival curve showing the association of vimentin expression with DFS, MFS, and OS. Statistical significance was evaluated using the log-rank test. The numbers below the curve represent the number of patients who are still at risk of experiencing the event (color coded to correspond to each group in the analysis).

Epithelial–mesenchymal transition is associated with the generation of CSCs [19]. Therefore, we evaluated the prognostic ability of vimentin in LA-NPC. Vimentin was expressed in 31% of the cases (Figure 3A, white arrow), and its expression was high in immune infiltrating cells (Figure 3A, black arrow). Importantly, vimentin expression in tumor cells significantly correlated with DFS (*p* = 0.016) but not with MFS or OS (Figure 3B). As shown in Supplementary Table S4, vimentin expression significantly correlated with CD24/CD44 coexpression (*p* = 0.004) but not with the expression of other CSC markers or clinicopathologic characteristics.

### 3.4. Low CD3^+^ TIL Density Is Associated with Shorter Survival

**Figure 4:**
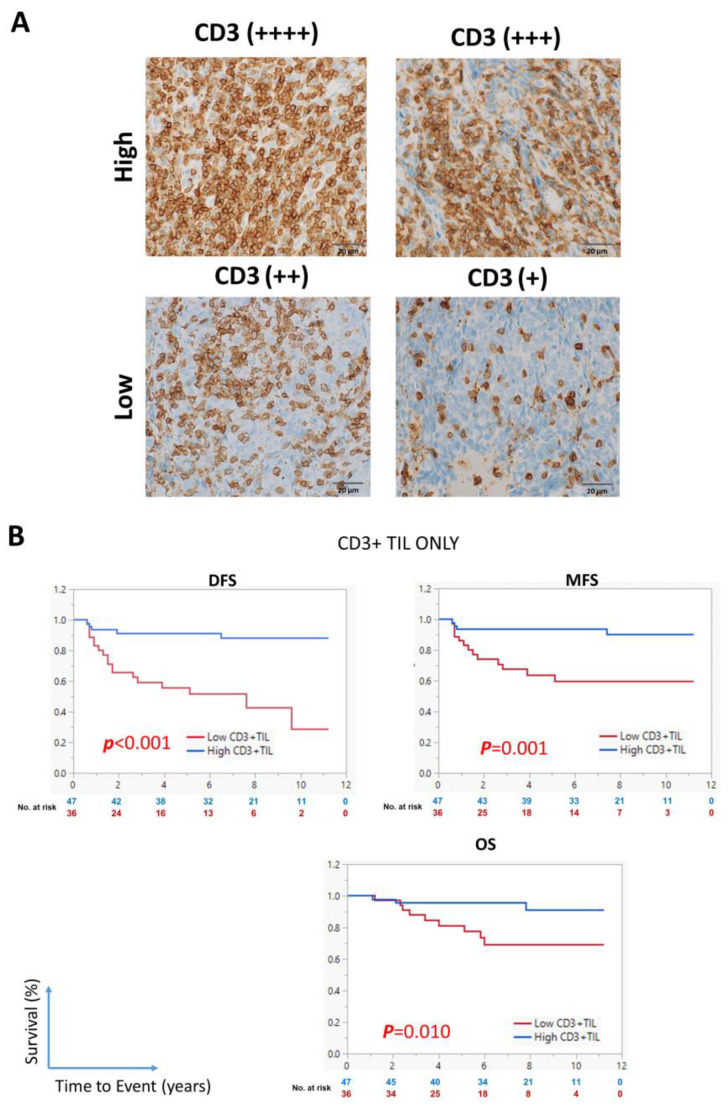
Prognostic ability of the CD3^+^ tumor-infiltrating lymphocyte density in patients with LA-NPC. (**A**) Representative images showing tissue specimens with high and low CD3^+^ TIL density (scores 3–4 and 1–2, respectively). (**B**) Kaplan–Meier survival curves showing the association of the CD3^+^ TIL density with DFS, MFS, and OS. Statistical significance was evaluated using the log-rank test. The numbers below the curve represent the number of patients who are still at risk of experiencing the event (color coded to correspond to each group in the analysis).

We re-evaluated the prognostic ability of the previously identified immune prognostic marker CD3^+^ TIL by comparing cases with low CD3^+^ TIL density (CD3^+^ TIL scores of 1–2) and those with high (CD3^+^ TIL score of 3–4) (Figure 4A). A low CD3^+^ TIL density significantly correlated with DFS (*p* < 0.001), MFS (*p* = 0.001), and OS (*p* = 0.010) (Figure 4B), in agreement with our previous reports [2,17]. Additionally, a low CD3^+^ TIL density significantly correlated with old age (*p* = 0.012) and high CD44 expression in tumor cells (*p* = 0.030) but not with the expression of other CSC markers or clinicopathologic characteristics (Supplementary Table S4).

### 3.5. CD24 Expression Is an Independent Prognostic Marker in LA-NPC

We used the Cox proportional hazards model to examine whether the prognostic ability of the CSC markers, including CD24, evaluated in the present study depended on other factors. The univariate Cox regression analysis of all CSC markers and vimentin confirmed that CD44, CD24, and CD44/CD24 expression significantly correlated with DFS (Table 1), and that CD24 expression, alone or in combination with CD44 expression, significantly correlated with OS. As reported in our previous study [11], the trial arm (the original different aim unrelated to this study) had no significant effect on the prognosis of patients with NPC (Supplementary Table S5). The univariate analysis of the clinicopathologic characteristics revealed that only the WHO histologic type correlated significantly with DFS (*p* = 0.005) and that the CD3^+^ TIL density significantly correlated with DFS (*p* < 0.001) and OS (*p* = 0.020) (Supplementary Table S5).

We then performed multivariate Cox regression analysis to determine factors independently associated with survival in patients with LA-NPC. To that end, all parameters that exhibited significant association with survival (DFS or OS) in the univariate analysis were included in the multivariate analysis. As shown in Table 2, the multivariate analysis revealed that only the CD3^+^ TIL density and the CD24 expression status significantly correlated with DFS and OS, although the correlation of the CD3^+^ TIL density with OS was borderline significant. The correlation of CD24 expression and the CD3^+^ TIL density with survival remained significant after removing other nonsignificant factors from the multivariate analysis (Table 3). To ensure that the missing values for the exhausted tissue blocks did not affect the results, we reran the multivariate analysis using the missing indicator method “informative missing option” in case the values were not missing at random. The multivariate analysis using the missing indicator method showed similar results (Supplementary Table S6), demonstrating that the missing values did not affect the conclusion. In addition, to ensure the previous trial with a different aim did not affect the results, we reran the multivariate analysis while including the trial arm. The multivariate analysis with the trial arm showed similar results, demonstrating that the trial arm did not affect the conclusion (Supplementary Table S7). Altogether, the univariate and multivariate Cox regression survival analyses indicated that CD24 expression and CD3^+^ TIL density were significant, independent prognostic factors for survival in patients with LA-NPC.

### 3.6. The Combination of CD24 Expression and CD3^+^ TIL Density Identifies High-Risk NPC Patients

**Figure 5:**
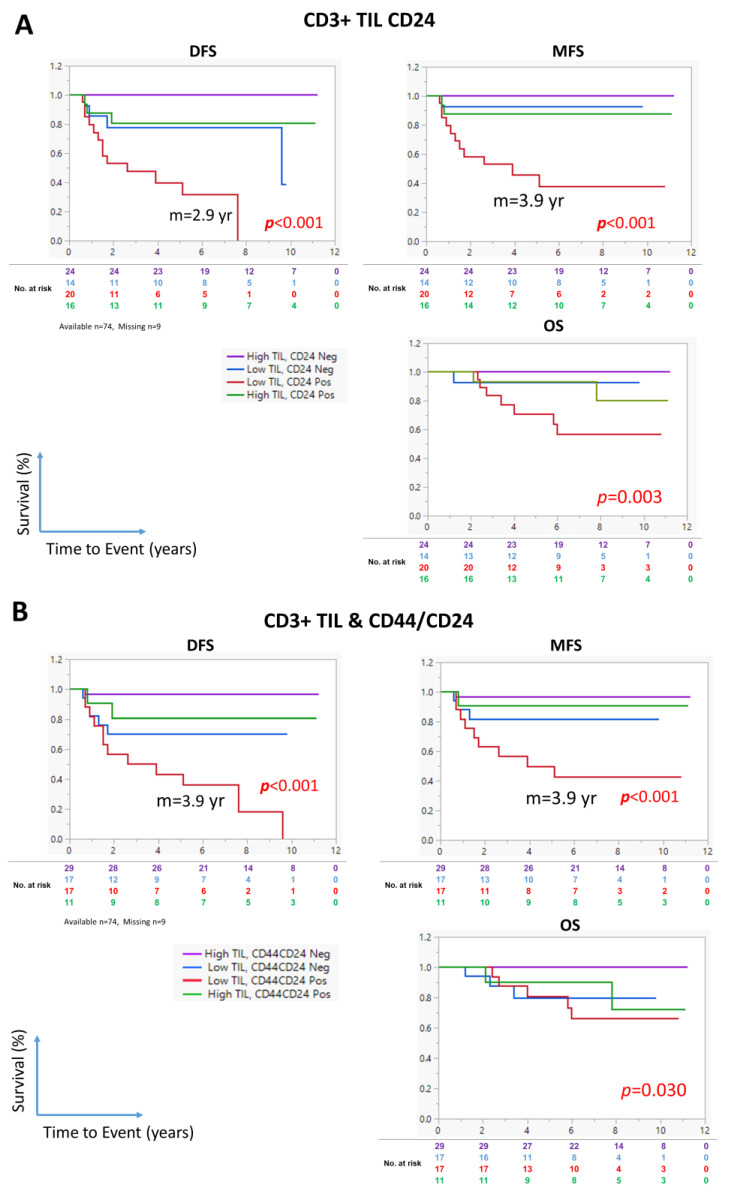
Prognostic ability of the CD3^+^ TIL density in combination with CD24 expression in tumor cells in patients with LA-NPC. Kaplan–Meier survival curves showing the association of the CD3^+^ TIL density and the expression of CD24, alone (**A**) or in combination with CD44 (**B**), with DFS, MFS, and OS. Statistical significance was evaluated using the log-rank test. Purple line, patients with high CD3^+^ TIL density and CD24-negative (**A**) or CD44/CD24 negative (**B**) NC; blue line, patients with low CD3^+^ TIL density and CD24-low (**A**) or CD44/CD24-negative (**B**) NPC; red line, patients with low CD3^+^ TIL density and CD24-high (A) or CD44/CD24-positive (**B**) NPC; green line, patients with high CD3^+^ TIL density and CD24-high (**A**) or CD44/CD24-positive (**B**) NPC. The numbers below the curve represent the number of patients who are still at risk of experiencing the event (color coded to correspond to each group in the analysis).

Finally, we evaluated the ability of the combined CD3^+^ TIL density and CD24 expression status to predict survival. As shown in Figure 5A, the patients with a low CD3^+^ TIL density and a high CD24 expression in tumor cells emerged as a subgroup with the shortest survival, with median DFS and MFS of 2.6 and 3.9 years, respectively (Figure 5A). The median survival for the other subgroups was undefined. Similarly, the patients with a low CD3^+^ TIL density and a high CD24/CD44 coexpression in tumor cells had the worst DFS, MFS, and OS, with a median of 3.9 years for both DFS and MFS (Figure 5B).

## 4. Discussion

Although NPC patients generally respond very well to CCRT, some relapse and experience short survival. In specific geographical locations, including Saudi Arabia, the majority of patients present with LA-NPC. Unfortunately, the prognostic power of TNM staging is limited in LA-NPC as all patients are, by definition, in TNM stage 3 or 4A according to American Joint Committee on Cancer classification. This is the first study to show the ability of the CSC marker CD24, alone or in combination with the CD3^+^ TIL density, to identify patients with significantly shorter DFS, MFS, and OS.

CSCs are a subset of tumor cells with self-renewal properties, higher rates of therapy resistance and disease metastasis. Several CSC markers were previously identified in NPC, including CD44 [7,20], ALDH1 [6,18], and the combination of CD44 and CD24 [8,21]. However, few studies have investigated the prognostic ability of the CSC markers in LA-NPC. For the first time, this study compares the prognostic ability of several CSC markers in LA-NPC, and our results reveal that only CD24 expression was an independent prognostic factor, as shown in the univariate and multivariate analyses.

In an in vitro work using the NPC cell lines 5–8F and CNE2, Wu et al. [18] demonstrated that cells with high ALDH1 expression had better tumor sphere-forming ability and formed larger tumors in immunocompromised NOD/SCID mice. The authors further showed that ALDH1 expression in NPC tissue was associated with worse survival. However, the studied cohort was composed of patients with NPC in different stages, including stage I and II NPC in 28% of the patients, and included both local and metastatic disease. Importantly, in their study, ALDH1 expression was associated with disease stage and metastatic status, which might explain its association with survival. Similarly, Luo et al. [22] reported the association between NPC overexpressing ALDH1 and shorter survival in a cohort of patients in varying disease stages, including stage I and II NPC in 33% of the patients. However, no study to date has evaluated the association of ALDH1 expression with prognosis in patients with higher-stage disease, such as those with LA-NPC, i.e., stage III or IV disease. In the present study, ALDH1 expression was not a significant prognostic marker in patients with LA-NPC.

CD44^+^ NPC cells were previously shown to exhibit higher proliferative ability and higher expression of the stemness antigens OCT4 and BMI1 [20]; they were also resistant to chemotherapy and radiotherapy. However, Janisiewicz et al. reported that CD44 was not a significant prognostic factor in NPC [23]. In the present study, our analyses showing the association of CD44 expression with DFS and MFS indicated its prognostic ability, although CD44 expression was not significantly associated with OS.

CD24 expression in NPC was initially recognized 30 years ago in a study reporting the association of CD24 expression with EBV-virus load [24]. Subsequent studies identified CD24 as a CSC marker in NPC [8] and its contribution to the reprogramming of NPC cells toward CSCs in coordination with CD44 [8]. However, this is the first study to demonstrate the ability of CD24 to predict DFS, MFS, and OS in patients with LA-NPC.

Ample evidence supports the association of CD24 expression with chemotherapy resistance, such as its association with doxorubicin and paclitaxel resistance in endometrial cancer cells [25], doxorubicin and cisplatin resistance in ovarian cancer cells [26], and gemcitabine resistance in pancreatic cancer cells [27]. In head and neck squamous cancer cells, CD24 expression has been reported to be associated with resistance to cisplatin [28], which could be reversed with CD24 inhibition. Moreover, CD24 expression was found to be associated with resistance to radiotherapy in head and neck squamous cancer cells. Specifically, CD24-positive NPC cells exhibited a higher proliferation rate, better ability to form tumorspheres, and increased chemotherapy resistance and tumor initiation in immunocompromised mice [8,9]. Interestingly, suppression of the Wnt/β-catenin-signaling pathway was shown to downregulate CD24 and abrogate CSCs and cisplatin resistance in NPC [29].

CD44^hi^/CD24^hi^ NPCs were previously reported to exhibit the characteristics of CSCs. Shen et al. [8] demonstrated that CD44^hi^/CD24^hi^ cells expressed higher levels of stemness genes and formed significantly more tumorspheres. Interestingly, CD44^hi^/CD24^hi^ cells exhibited a mesenchymal-type morphology and expressed higher levels of vimentin, consistent with our finding that CD44/CD24 coexpression in tumor cells was associated with vimentin-positive tumors. Importantly, we also found that high CD44/CD24 coexpression in tumor cells was associated with worse DFS, MFS, and OS.

Several studies demonstrated the relationship between CSCs and immune response. CSCs play an important role in the recruitment of tumor-associated macrophages, which promote CSC expansion. On the other hand, CSCs can escape natural killer (NK) cells by downregulating the NKG2D ligands MICA and MICB [30]. Moreover, CSCs can avoid TIL via the overexpression of PD-L1 overexpression [31,32]. However, evidence regarding the combinations of CSC and immune markers as prognostic tools is limited. This is the first study to show that the combination of a low CD3^+^ TIL density and a positive CD24 expression in LA-NPC was significantly associated with shorter survival. Remarkably, the CSC marker status was not associated with DFS or MFS in NPC patients with a high CD3^+^ TIL density. Conversely, the CSC marker status was crucial in NPC patients with a low CD3^+^ TIL density. Combining immune- and CSC-related markers introduces a novel concept in cancer prognosis and holds promise in enhancing our understanding of cancer subpopulations and guiding personalized therapeutic strategies.

In humans, several CD24 ligands have been identified, including siglec-10 expressed in immune cells such as monocytes/macrophages and B-cells, p-selectin expressed in endothelial cells, and L1-CAM expressed in leukocytes. The CD24/siglec-10 interaction has contrasting roles in different immune cells, acting in a costimulatory capacity in T cells [33] and in an inhibitory capacity in other immune cells such as NK cells [34] and phagocytes [35]. This dichotomy underlies the complex role of CD24 in tumor immunology. It is possible that in NPCs with low CD3^+^ TIL density, the immune-stimulatory role of tumoral CD24 is outweighed by its inhibitory effect due to the interaction with siglec-10 in macrophages/phagocytes and NK cells, tipping the balance towards a dominantly inhibitory function of CD24, which should be elucidated in future studies.

CD3^+^ TIL density and CD24 expression, alone or in combination with CD44 expression, were significantly associated with RFS, MFS and OS. However, the association of the combined CD3^+^ TIL density and CD24 expression status with MFS is probably the most critical in patients with LA-NPC, as local relapse can be retreated with CCRT, while managing LA-NPC is much more challenging once it becomes metastatic. Therefore, identifying high-risk patients that are likely to become metastatic might be the most crucial, especially if an alternative or more aggressive therapy is available. Further work is needed to test this.

It is important to note the main study limitations: the retrospective design and the inclusion of patients initially recruited for a different study. These factors might have introduced confounding factors unrelated to the present study. However, it is reassuring to know that the factors unrelated to the goal of the present study (trial arms) did not have a significant effect on survival. Furthermore, the administration of a standardized treatment regimen in all patients was a particular strength of our study. Other limitations for this study include the use of a single cohort with a possibility of not being fully representative for LA-NPC cases, in addition to having missing values for nine cases for CD24, CD44 and ALDH1 testing. These limitations suggest that the findings should be validated in another cohort.

## 5. Conclusions

Among the several CSC makers evaluated, CD24 was the only significant and independent prognostic marker for LA-NPC. The combination of the CD3^+^ TIL density and CD24 expression status identified a subset of patients with the shortest survival, which warrants further investigation.

## Figures and Tables

**Table 1:** Univariate Cox proportional hazard regression analysis of markers of CSCs and EMT with disease-free survival (DFS) and overall survival (OS) in 83 patients with LA-NPC.

	Relapse		DFS			Death		OS		
	−	+	HR	95% CI	* p	−	+	HR	95% CI	* p
**BMI1**										
Neg (score 1 or 2)	22 (76)	7 (24)	1			26 (90)	3 (10)	1		
Pos (score 3 or 4)	37 (70)	16 (30)	1.2	0.5–2.9	0.700	44 (83)	9 (17)	1.4	0.4–5.3	0.596
**^◊^ CD44**										
Neg (<70%)	41 (84)	8 (16)	1			44 (90)	5 (10)	1		
Pos (≥70%)	13 (52)	12 (48)	3.2	1.3–7.9	**0.011**	20 (80)	5 (20)	1.9	0.6–6.7	0.298
**^◊^ ALDH1**										
Neg (<10%)	15 (83)	3 (17)	1			33 (87)	5 (13)	1		
Pos (≥10%)	39 (70)	17 (30)	2.0	0.6–7.0	0.256	31 (86)	5 (14)	1.4	0.3–6.7	0.659
**^◊^ ALDH1/CD44**										
Neg (<10%)	30 (81)	7 (19)	1			37 (86)	6 (14)	1		
Pos (≥10%)	24 (65)	13 (35)	1.8	0.7–4.5	0.211	27 (87)	4 (13)	0.9	0.3–3.2	0.932
**^◊^ CD24**										
Neg (<30%)	33 (89)	4 (11)	1			43 (93)	3 (7)	1		
Pos (≥30%)	21 (57)	16 (43)	5.3	1.8–16.0	**0.003**	21 (75)	7 (25)	10.8	1.4–85.5	**0.024**
**^◊^ CD24/CD44**										
Neg (<10%)	40 (87)	6 (13)	1			43 (93)	3 (7)	1		
Pos (≥10%)	14 (50)	14 (50)	4.6	1.7–11.9	**0.002**	21 (75)	7 (25)	4.0	1.0–15.6	**0.044**
**Vimentin**										
Neg (score 1 or 2)	46 (81) ^♣^	11 (19)	1			51 (89)	6 (11)	1		
Pos (score 3 or 4)	14 (54)	12 (46)	2.6	1.2–6.0	**0.021**	20 (77)	6 (23)	2.4	0.8–7.4	0.134

**Abbreviations**: Neg = negative, Pos = positive, (+ and −) are numbers of patients, * *p* values in bold represent significant data, ^♣^ Numbers between brackets are the percentages of patients. One sample is missing from BMI1. **^◊^** Nine samples are missing from CD44, CD24, ALDH1, ALDH1/CD44 and CD24/CD44.

**Table 2:** Multivariate Cox proportional hazard regression analysis of the different CSCs markers with disease-free survival (DFS) and overall survival (OS) in 83 patients with LA-NPC.

	Relapse		DFS			Death		OS		
	−	+	HR	95% CI	* p	−	+	HR	95% CI	* p
**WHO Type**										
III	59 (76)	19 (24)	1			67 (86)	11 (14)	1		
I and II	1 (20) ^♣^	4 (80)	3.2	0.9–11.7	0.080	4 (80)	1 (20)	2.1	0.2–21.8	0.541
**Vimentin**										
Negative	46 (81)	11 (19)	1			51 (89)	6 (11)	1		
Positive	14 (54)	12 (46)	1.1	0.4–3.2	0.819	20 (77)	6 (23)	1.3	0.3–5.3	0.714
**^◊^ CD44**										
<70%	41 (84)	8 (16)	1			44 (90)	5 (10)	1		
≥70%	13 (52)	12 (48)	2.0	0.7–5.1	0.178	20 (80)	5 (20)	1.0	0.2–3.7	0.952
**^◊^ CD24**										
<30%	33 (89)	4 (11)	1			36 (97)	1 (3)	1		
≥30%	21 (57)	16 (43)	4.3	1.3–13.9	**0.015**	28 (76)	9 (24)	8.9	1.1–73.0	**0.041**
**CD3^+^ TIL**										
High	42 (89)	5 (11)	1			44 (94)	3 (6)	1		
Low	18 (50)	18 (50)	7.4	1.9–28.6	**0.004**	27 (75)	9 (25)	4.9	0.9–26.9	0.068

**Abbreviations**: (+ and −) are numbers of patients, * *p* values in bold and shaded represent significant data, ^♣^ numbers between brackets are the percentages of patients, **^◊^** 9 samples are missing from CD44, and CD24 data.

**Table 3:** Multivariate Cox proportional hazard regression analysis of the different CSCs markers with disease-free survival (DFS) and overall survival (OS) in 83 patients with LA-NPC.

	Relapse		DFS			Death		OS		
	−	+	HR	95% CI	* p	−	+	HR	95% CI	* p
**^◊^ CD24**										
Negative	33 (89)	4 (11)	1			36 (97)	1 (3)	1		
Positive	21 (57)	16 (43)	4.8	1.6–15.0	**0.007**	28 (76)	9 (24)	8.6	1.1–69.0	**0.042**
**CD3^+^ TIL**										
High	42 (89)	5 (11)	1			44 (94)	3 (6)	1		
Low	18 (50)	18 (50)	11.1	2.7–35.0	**<0.001**	27 (75)	9 (25)	4.6	1.0–22.0	0.057

**Abbreviations**: * *p* values in bold and shaded represent significant data, **^◊^** 9 samples are missing from CD24 data.

## Data Availability

The analyzed datasets in this study are included in this published article (or Supplementary Files) and are available from the corresponding author upon reasonable request.

## Supplementary Materials

The following supporting information can be downloaded at: https://www.mdpi.com/article/10.3390/cancers17132094/s1, Figure S1: Representative images of antibody validation; Figure S2: Receiver Operating Characteristic (ROC) curve and Youden Index analysis; Figure S3: ALDH1 and CD24 expression in immune-infiltrating cells; Figure S4: Representative images of ALDH1 and CD24 different staining intensities in NPC sections; Figure S5: Prognostic ability of ALDH1 and CD24 expression based on H-scores; Table S1: Patients Characteristics; Table S2: Correlation between BMI1 and ALDH1 expression with other CSC makers; Table S3: Correlation between CD44, CD24, or CD44/CD24 combination and the expression of other CSC makers; Table S4: Correlation between CD3^+^ TIL and Vimentin expression with other CSC makers; Table S5: Univariate Cox proportional hazard regression analysis of the different clinicopathological features; Table S6: Multivariate Cox proportional hazard regression analysis using the “informative missing” option; Table S7: Multivariate Cox proportional hazard regression analysis while including the different aim trial arm.

## Abbreviations

CCRT	concurrent chemoradiotherapy
DFS	disease-free survival
LA	locally advanced
MFS	metastasis-free survival
NK	natural killer
NPC	nasopharyngeal carcinoma
OS	overall survival
TIL	tumor-infiltrating lymphocytes
