# Supplementary material for: High Tumoral CD24 Expression and Low CD3+ Tumor-Infiltrating Lymphocytes as a Biomarker for High-Risk Locally Advanced Nasopharyngeal Carcinoma
Source: Cancers (Basel). 2025 Jun 23;17(13):2094. doi: 10.3390/cancers17132094 (PMC12249431; doi:10.3390/cancers17132094)
Supplement: Supplementary file 1 [file cancers-17-02094-s001.zip › Supplementary Table S1.pdf]

Supplementary Table S1. Patients Characteristics.

Features	Categories	# Patients
**Age**<br>Range 18-72<br>Median 45	< 40 years<br>≥ 40 years	32 *(39)<br>51 (61)
**Gender**	Male<br>Female	62 (75)<br>21 (25)
**WHO Type**	I<br>II<br>III	0 (0)<br>5 (6)<br>78 (94)
**T stage**	T1<br>T2<br>T3<br>T4	26 (31)<br>2 (2)<br>30 (36)<br>25 (30)
**N stage**	N0<br>N1<br>N2<br>N3	5 (6)<br>12 (15)<br>24 (29)<br>42 (51)
**TNM Staging**	III<br>IV	24 (29)<br>59 (71)
**Relapse**	No<br>Yes	60 (72)<br>23 (28)
**Type of Relapse**	None<br>Local<br>Locoregional<br>Systemic	60 (72)<br>3 (4)<br>3 (4)<br>17 (20)
**Survival**	Alive<br>Dead	71 (86)<br>12 (14)

* Percentage of cases
