# Supplementary material for: High Tumoral CD24 Expression and Low CD3+ Tumor-Infiltrating Lymphocytes as a Biomarker for High-Risk Locally Advanced Nasopharyngeal Carcinoma
Source: Cancers (Basel). 2025 Jun 23;17(13):2094. doi: 10.3390/cancers17132094 (PMC12249431; doi:10.3390/cancers17132094)
Supplement: Supplementary file 1 [file cancers-17-02094-s001.zip › Supplementary Table S2.pdf]

Supplementary Table S2. Correlation between BMI1 and ALDH1 expression with other CSC makers and clinicopathological parameters of 83 LA-NPC patients.

	BMI1			ALDH1 (≥ 10%)			ALDH1/CD44 (≥ 10%)		
	-	+	**p*	-	+	**p*			**p*
**Age**									
< 40 years	12 (37)	20 (63)	0.815	6 (21)	23 (79)	0.594	16 (55)	13 (45)	0.634
≥ 40 years	17 (34)	33 (66)		12 (27)	33 (73)		21 (47)	24 (53)	
**Gender**									
Male	21 (34)	40 (66)	0.795	12 (21)	44 (79)	0.350	27 (48)	29 (52)	0.787
Female	8 (38)	13 (62)		6 (33)	12 (67)		10 (56)	8 (44)	
**WHO Type**									
I & II	2 (40)	3 (60)	1.000	2 (40)	3 (60)	0.590	4 (80)	1 (20)	0.358
III	27 (35)	50 (65)		16 (23)	53 (77)		33 (47)	36 (52)	
**T stage**									
I & II	11 (39)	17 (61)	0.632	6 (25)	18 (75)	1.000	13 (54)	11 (46)	0.804
III & IV	18 (33)	36 (67)		12 (24)	38 (76)		24 (48)	26 (52)	
**N stage**									
N0 & N1	5 (31)	11 (69)	0.778	4 (29)	10 (71)	0.734	6 (43)	8 (57)	0.768
N2 & N3	24 (37)	42 (64)		14 (23)	46 (77)		31 (52)	29 (48)	
**Disease Stage (UICC)**									
III	8 (35)	15 (65)	1.000	3 (16)	16 (84)	0.372	9 (47)	10 (53)	1.000
IVA	21 (36)	38 (64)		15 (27)	40 (73)		28 (51)	27 (49)	
**#BMI1**									
Negative				9 (33)	18 (67)	0.260	18 (67)	9 (33)	0.052
Positive				9 (19)	38 (81)		19 (40)	28 (60)	
**#ALDH1**									
< 10%	18 (47)	20 (53)	0.561						
≥ 10%	9 (25)	27 (75)							
**#CD44**									
< 70%	19 (39)	30 (61)	0.618	13 (27)	36 (73)	0.582			
≥ 70%	8 (32)	17 (68)		5 (20)	20 (80)				
**#CD24**									
< 30%	18 (39)	28 (61)	0.623	12 (32)	25 (68)	0.175	21 (57)	16 (43)	0.353
≥ 30%	9 (32)	19 (68)		6 (16)	31 (84)		16 (43)	21 (57)	
**#CD44/CD24**									
< 10%	18 (39)	28 (61)	0.623	**15 (33)**	**31 (67)**	**0.049**			
≥ 10%	9 (32)	19 (68)		**3 (11)**	**25 (89)**				
**Trial Arm**									
LDXRT	15 (37)	25 (63)	0.818	8 (22)	28 (78)	0.789	18 (50)	18 (50)	1.000
Control arm	14 (33)	28 (67)		10 (26)	28 (74)		19 (50)	19 (50)	

Abbreviations: **p* values in bold and highlighted represent significant data. 1 Sample is missing from BMI1.
◊ 9 Samples are missing from ALDH1, CD44, CD24 and CD24/CD44 data. Highlighted empty areas are for the marker with itself.
