# Supplementary material for: High Tumoral CD24 Expression and Low CD3+ Tumor-Infiltrating Lymphocytes as a Biomarker for High-Risk Locally Advanced Nasopharyngeal Carcinoma
Source: Cancers (Basel). 2025 Jun 23;17(13):2094. doi: 10.3390/cancers17132094 (PMC12249431; doi:10.3390/cancers17132094)
Supplement: Supplementary file 1 [file cancers-17-02094-s001.zip › Supplementary Table S3.pdf]

Supplementary Table S3. Correlation between CD44, CD24, CD44/CD24 combination and the expression of other CSC makers and clinicopathological parameters of 83 LA-NPC patients.

	CD44 (≥ 70%)			CD24 (≥ 30%)			CD44+/CD24+ (≥ 10%)		
	-	+	*p	-	+	*p	-	+	*p
**Age**									
< 40 years	**26 (90)**	**3 (10)**	**<0.001**	14 (48)	15 (52)	1.000	18 (62)	11 (38)	1.000
≥ 40 years	**23 (51)**	**22 (49)**		23 (51)	22 (49)		28 (62)	17 (38)	
**Gender**									
Male	36 (64)	20 (36)	0.582	31 (55)	25 (45)	0.175	36 (64)	20 (36)	0.581
Female	13 (72)	5 (28)		6 (33)	12 (67)		10 (56)	8 (44)	
**WHO Type**									
I & II	3 (60)	2 (40)	1.000	2 (40)	3 (60)	1.000	2 (40)	3 (60)	0.360
III	46 (67)	23 (33)		35 (51)	34 (49)		44 (64)	25 (36)	
**T stage**									
I & II	**20 (83)**	**4 (17)**	**0.038**	13 (54)	11 (46)	0.804	16 (67)	8 (33)	0.619
III & IV	**29 (58)**	**21 (42)**		24 (48)	26 (52)		30 (60)	20 (40)	
**N stage**									
N0 & N1	7 (50)	7 (50)	0.216	8 (57)	6 (43)	0.768	8 (57)	6 (43)	0.763
N2 & N3	42 (70)	18 (30)		29 (48)	31 (52)		38 (63)	22 (37)	
**Disease Stage (UICC)**									
III	10 (53)	9 (47)	0.168	11 (58)	8 (42)	0.600	11 (58)	8 (42)	0.785
IVA	39 (71)	16 (29)		26 (47)	29 (53)		35 (64)	20 (36)	
**#BMI1**									
Negative	19 (70)	8 (30)	0.618	14 (52)	13 (48)	1.000	18 (67)	9 (33)	0.623
Positive	30 (64)	17 (36)		23 (49)	24 (51)		28 (60)	19 (40)	
**#ALDH1**									
< 10%	13 (72)	5 (28)	0.582	12 (67)	6 (33)	0.175	**15 (83)**	**3 (17)**	**0.049**
≥ 10%	36 (64)	20 (36)		25 (45)	31 (55)		**31 (55)**	**25 (45)**	
**#CD44**									
< 70%				24 (49)	25 (51)	1.000			
≥ 70%				13 (52)	12 (48)				
**#CD24**									
< 30%	24 (65)	13 (35)	1.000						
≥ 30%	25 (68)	12 (32)							
**Trial Arm**									
LDXRT	24 (67)	12 (33)	1.000	20 (56)	16 (44)	0.486	24 (67)	12 (33)	0.480
Control arm	25 (66)	13 (34)		17 (45)	21 (55)		22 (58)	16 (42)	

Abbreviations: **p* values in bold and highlighted represent significant data. 1 Sample is missing from BMI1.
◊ 9 Samples are missing from ALDH1, CD44, CD24 and CD24/CD44 data. Highlighted empty areas are for the marker with itself.
