# Supplementary material for: High Tumoral CD24 Expression and Low CD3+ Tumor-Infiltrating Lymphocytes as a Biomarker for High-Risk Locally Advanced Nasopharyngeal Carcinoma
Source: Cancers (Basel). 2025 Jun 23;17(13):2094. doi: 10.3390/cancers17132094 (PMC12249431; doi:10.3390/cancers17132094)
Supplement: Supplementary file 1 [file cancers-17-02094-s001.zip › Supplementary Table S4.pdf]

Supplementary Table S4. Correlation between CD3+TIL and Vimentin expression with other CSC makers and clinicopathological parameters of 83 LA-NPC patients.

	Vimentin			CD3+TIL		
	-	+	**p*	-	+	**p*
**Age**						
< 40 years	12 (37)	20 (63)	0.815	**8 (25)**	**24 (75)**	**0.012**
≥ 40 years	17 (34)	33 (33)		**28 (55)**	**23 (45)**	
**Gender**						
Male	43 (69)	19 (31)	0.793	27 (44)	35 (56)	1.000
Female	14 (67)	7 (33)		9 (43)	12 (57)	
**WHO Type**						
I & II	2 (40)	3 (60)	0.175	3 (60)	2 (40)	0.648
III	55 (71)	23 (29)		33 (42)	45 (58)	
**T stage**						
I & II	18 (64)	10 (36)	0.619	9 (32)	19 (68)	0.165
III & IV	39 (71)	16 (29)		27 (49)	28 (51)	
**N stage**						
N0 & N1	13 (76)	4 (24)	0.563	8 (47)	9 (53)	0.788
N2 & N3	44 (67)	22 (33)		28 (42)	38 (58)	
**Disease Stage (UICC)**						
III	16 (67)	8 (33)	0.800	13 (54)	11 (46)	0.230
IVA	41 (69)	18 (31)		23 (39)	36 (61)	
**Vimentin**						
Negative				24 (42)	33 (58)	0.813
Positive				12 (46)	14 (54)	
**#BMI1**						
Negative	18 (62)	11 (38)	0.458	12 (41)	17 (59)	0.818
Positive	38 (72)	15 (28)		24 (45)	29 (55)	
**#ALDH1**						
< 10%	11 (61)	7 (39)	0.380	5 (28)	13 (72)	0.104
≥ 10%	41 (73)	15 (27)		29 (52)	27 (48)	
**#CD44**						
< 70%	36 (73)	13 (27)	0.430	**18 (37)**	**31 (63)**	**0.030**
≥ 70%	16 (64)	9 (36)		**16 (64)**	**9 (36)**	
**#CD24**						
< 30%	29 (79)	8 (22)	0.203	14 (38)	23 (62)	0.243
≥ 30%	23 (62)	14 (38)		20 (54)	17 (45)	
**#CD44/CD24**						
< 10%	**38 (83)**	**8 (17)**	**0.004**	17 (37)	29 (63)	0.057
≥ 10%	**14 (50)**	**14 (50)**		17 (61)	11 (39)	
**CD3+ TIL**						
Low	24 (67)	12 (33)	0.813			
High	33 (70)	14 (30)				
**Trial Arm**						
LDXRT	29 (71)	12 (29)	0.814	22 (54)	19 (46)	0.078
Control arm	28 (67)	14 (33)		14 (33)	28 (67)	

Abbreviations: **p* values in bold and highlighted represent significant data. 1 Sample is missing from BMI1. ◊ 9 Samples are missing from ALDH1, CD44, CD24 and CD24/CD44 data. Highlighted empty areas are for the marker with itself.
