# Supplementary material for: High Tumoral CD24 Expression and Low CD3+ Tumor-Infiltrating Lymphocytes as a Biomarker for High-Risk Locally Advanced Nasopharyngeal Carcinoma
Source: Cancers (Basel). 2025 Jun 23;17(13):2094. doi: 10.3390/cancers17132094 (PMC12249431; doi:10.3390/cancers17132094)
Supplement: Supplementary file 1 [file cancers-17-02094-s001.zip › Supplementary Table S5.pdf]

Supplementary Table S5. Univariate Cox proportional hazard regression analysis of the different clinicopathological features and biomarkers with disease-free survival (DFS) and Overall Survival (OS) in 83 patients with LA-NPC.

	Relapse		DFS			Death		OS		
	**-**	**+**	**HR**	**95% CI**	***p**	**-**	**+**	**HR**	**95% CI**	***p**
**Age**										
< 40 years	27 (84)	5 (16)	1			29 (91)	3 (9)	1		
≥ 40 years	33 (65)	18 (35)	2.5	1.0-6.8	0.067	42 (82)	9 (18)	1.9	0.5-7.2	0.320
**Gender**										
Male	43 (69)	19 (31)	1			51 (82)	11 (18)	1		
Female	17 (81)	4 (19)	0.5	0.2-1.6	0.256	20 (95)	1 (5)	0.2	0.03-1.9	0.174
**WHO Type**										
III	**59 (76)**	**19 (24)**	**1**			67 (86)	11 (14)	1		
I & II	**1 (20)**	**4 (80)**	**4.8**	**1.6-14.4**	**0.005**	4 (80)	1 (20)	1.1	0.1-8.5	0.930
**T stage**										
I & II	22 (79)	6 (21)	1			26 (93)	2 (7)	1		
III & IV	38 (69)	17 (31)	1.7	0.7-4.3	0.278	45 (82)	10 (18)	3.0	0.7-13.8	0.157
**N stage**										
N0 & N1	13 (76)	4 (24)	1			15 (88)	2 (12)	1		
N2 & N3	47 (71)	19 (29)	1.3	0.4-3.9	0.612	56 (85)	10 (15)	1.4	0.3-6.2	0.698
**Disease Stage (UICC)**										
III	17 (71)	7 (29)	1			20 (83)	4 (17)	1		
IVA	43 (73)	16 (27)	0.9	0.4-2.2	0.797	51 (86)	8 (14)	0.8	0.2-2.5	0.656
**CD3+TIL**										
High	42 (89)	5 (11)	**1**			**44 (94)**	**3 (6)**	**1**		
Low	18 (50)	18 (50)	**6.5**	**2.4-17.8**	**< 0.001**	**27 (75)**	**9 (25)**	**4.8**	**1.3-17.7**	**0.020**
**Trial Arm ♠**										
Control arm	32 (76)	10 (24)	1			35 (83)	7 (17)	1		
LDXRT	28 (68)	13 (32)	1.5	0.7-3.5	0.313	36 (88)	5 (12)	1.27	0.4-4.0	0.688

Abbreviations: Neg=Negative, Pos=Positive, (+ and -) are numbers patients, **p* values in bold represent significant data, *Numbers between brackets are the percentages of patients. ♠ Control arm = No irradiation during neoadjuvant chemotherapy, LDXRT =Lose dose of irradiation during the neoadjuvant chemotherapy.
