# Supplementary material for: High Tumoral CD24 Expression and Low CD3+ Tumor-Infiltrating Lymphocytes as a Biomarker for High-Risk Locally Advanced Nasopharyngeal Carcinoma
Source: Cancers (Basel). 2025 Jun 23;17(13):2094. doi: 10.3390/cancers17132094 (PMC12249431; doi:10.3390/cancers17132094)
Supplement: Supplementary file 1 [file cancers-17-02094-s001.zip › Supplementary Table S6.pdf]

Supplementary Table S6. Multivariate Cox proportional hazard regression analysis of the different CSCs markers with disease-free survival (DFS) and overall Survival (OS) in 83 patients with LA-NPC using the "informative missing" option.

	DFS	OS
	***p**	***p**
**WHO Type**		
III		
I & II	0.117	0.438
**Vimentin**		
Negative		
Positive	0.413	0.259
**CD44**		
< 70%		
≥ 70%	0.041	0.137
**CD24**		
< 30%		
≥ 30%	**0.021**	**0.046**
**CD3+TIL**		
High		
Low	**0.005**	**0.047**

Abbreviations: (+ and -) are numbers patients, **p* values in bold and shaded represent significant data.
