# Supplementary material for: High Tumoral CD24 Expression and Low CD3+ Tumor-Infiltrating Lymphocytes as a Biomarker for High-Risk Locally Advanced Nasopharyngeal Carcinoma
Source: Cancers (Basel). 2025 Jun 23;17(13):2094. doi: 10.3390/cancers17132094 (PMC12249431; doi:10.3390/cancers17132094)
Supplement: Supplementary file 1 [file cancers-17-02094-s001.zip › Supplementary Table S7.pdf]

Supplementary Table S7. Multivariate Cox proportional hazard regression analysis of the different CSCs markers with disease-free survival (DFS) and overall Survival (OS) in 83 patients with LA-NPC.

	DFS	OS
	***p**	***p**
**WHO Type**		
III		
I & II	0.060	0.402
**Vimentin**		
Negative		
Positive	0.678	0.440
**CD44**		
< 70%		
≥ 70%	0.129	0.483
**CD24**		
< 30%		
≥ 30%	**0.013**	**0.033**
**CD3+TIL**		
High		
Low	**0.008**	**0.019**
**Trial Arm**		
High		
Low	0.507	0.081

Abbreviations: (+ and -) are numbers patients, *p values in bold and shaded represent significant data.
