# Supplementary figures and images for: High Tumoral CD24 Expression and Low CD3+ Tumor-Infiltrating Lymphocytes as a Biomarker for High-Risk Locally Advanced Nasopharyngeal Carcinoma
Source: Cancers (Basel). 2025 Jun 23;17(13):2094. doi: 10.3390/cancers17132094 (PMC12249431; doi:10.3390/cancers17132094)

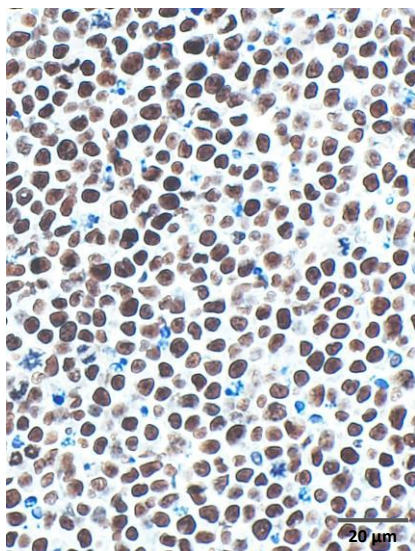

U-937

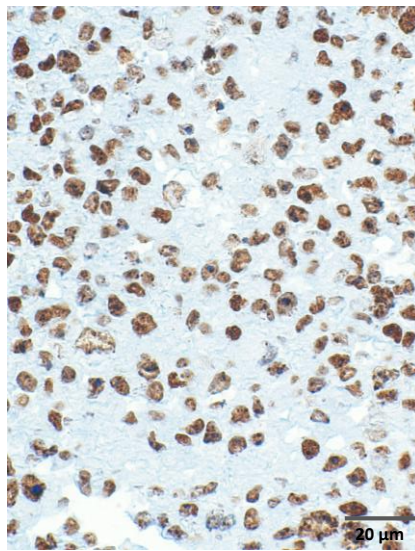

MDA-MB-231

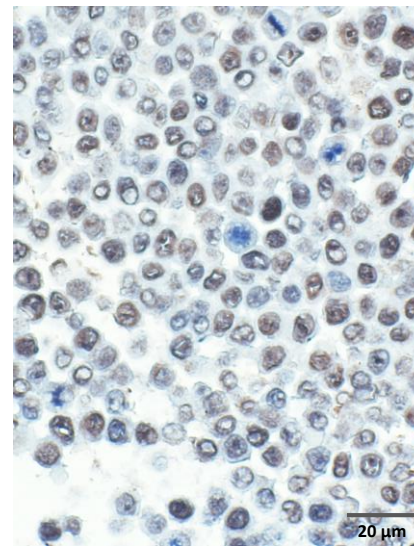

MDA-MB-468

**BMI1**

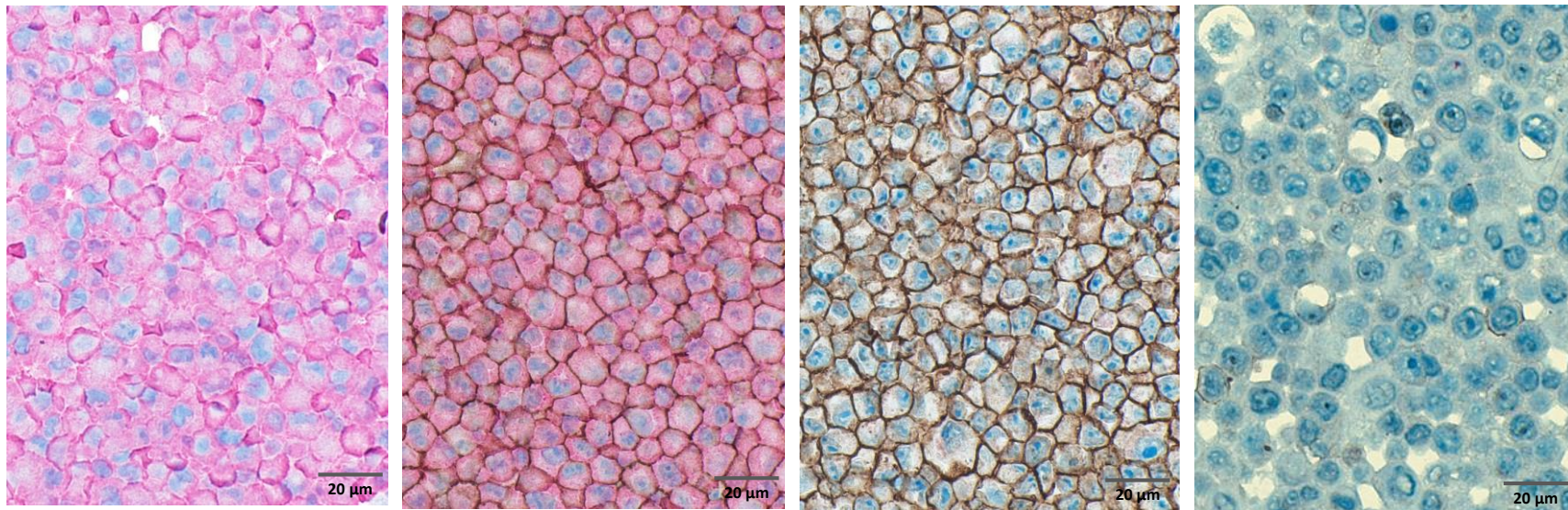

A549

A549

MDA-MB-231

MCF-7

ALDH1

ALDH1/CD44

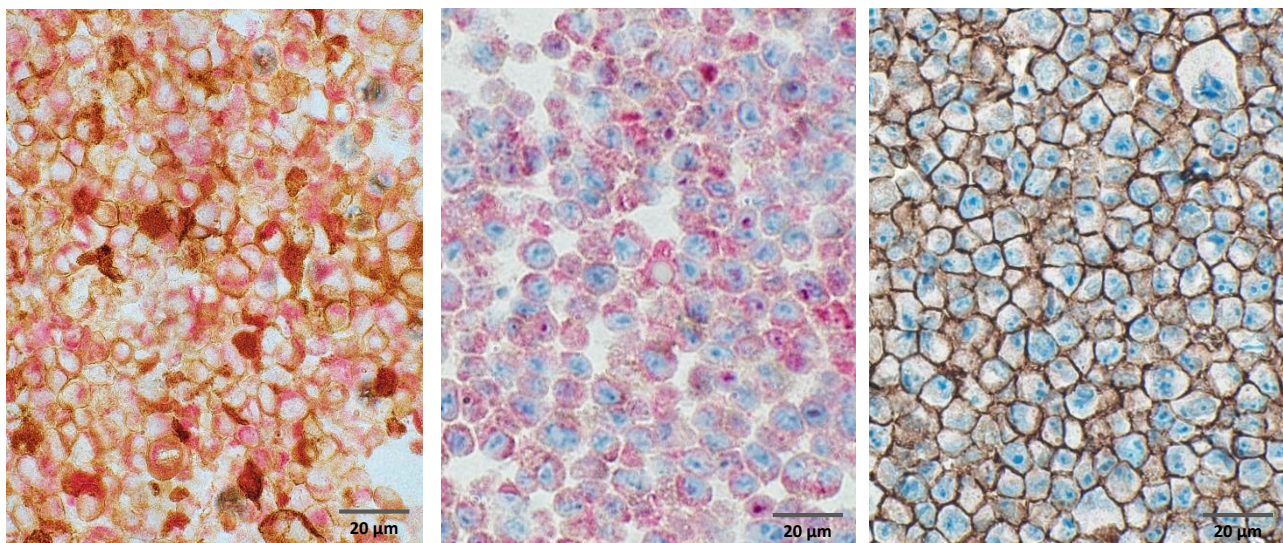

SUM149PT

SK-BR-3

MDA-MB-231

CD24/CD44

Supplement: Supplementary file 1 [file cancers-17-02094-s001.zip › Supplementary Figure S1.pdf]

CD44

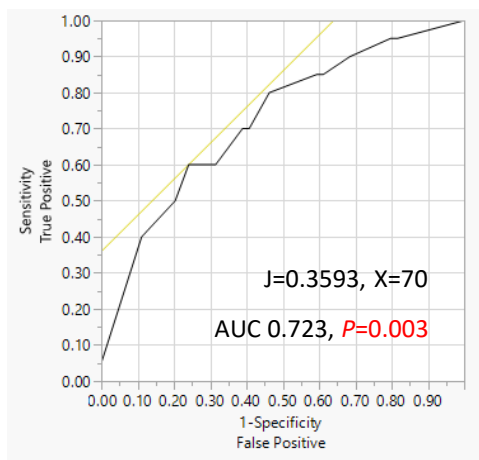

CD24

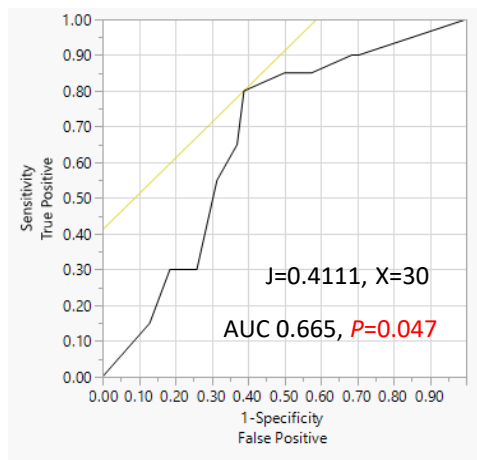

CD44/CD24

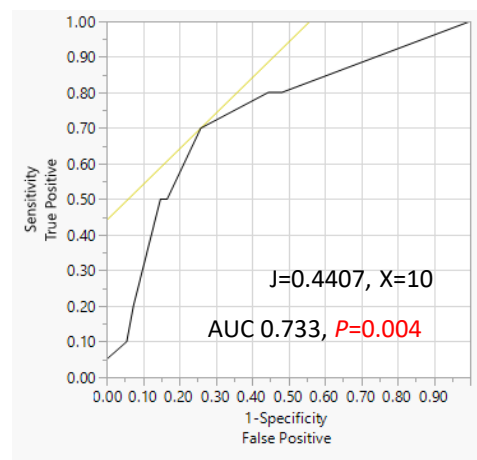

ALDH

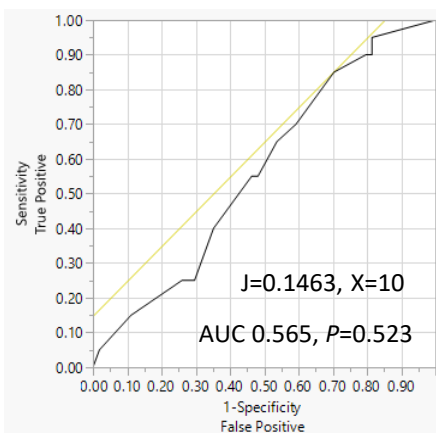

ALDH/CD44

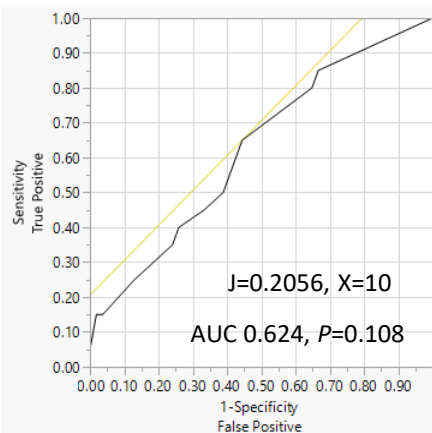

ALDH Score

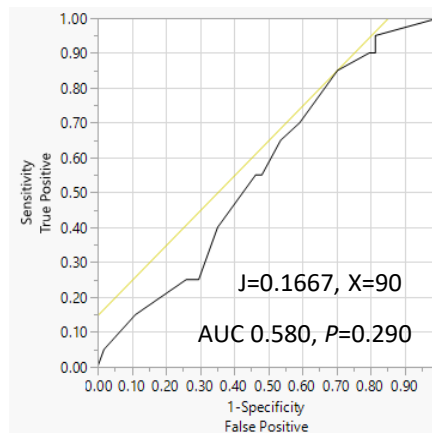

CD24 Score

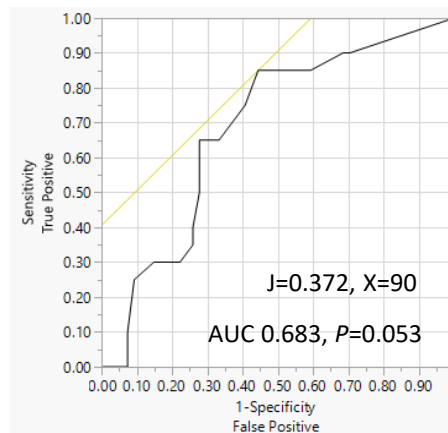

Supplement: Supplementary file 1 [file cancers-17-02094-s001.zip › Supplementary Figure S2.pdf]

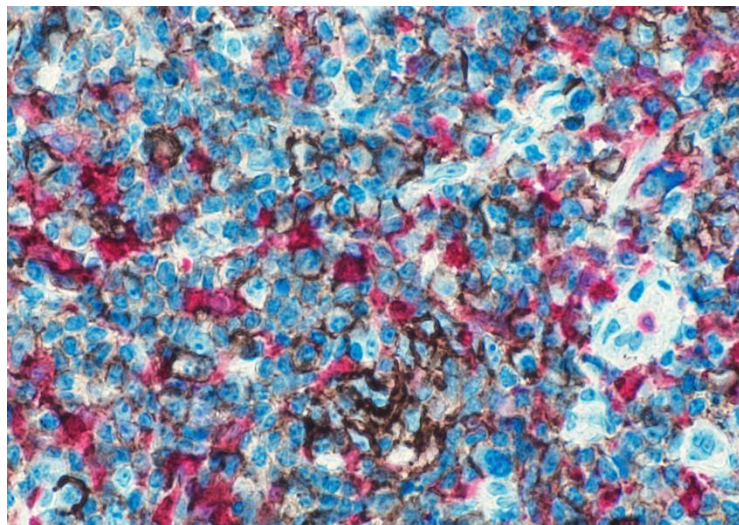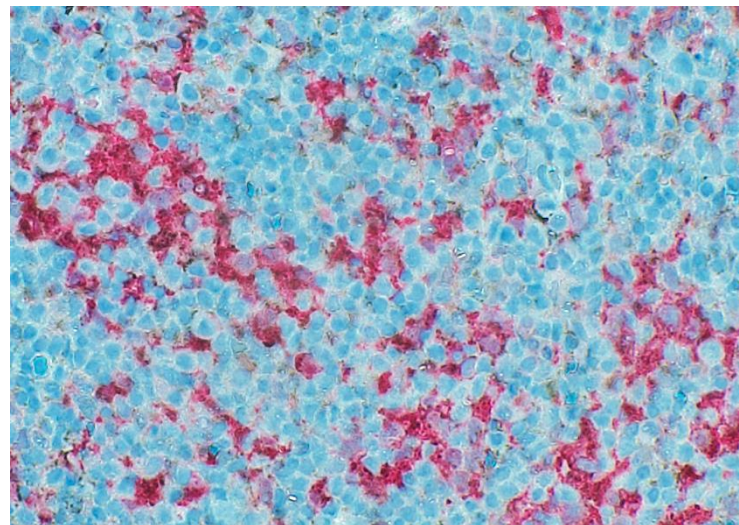

ALDH1 in immune cells

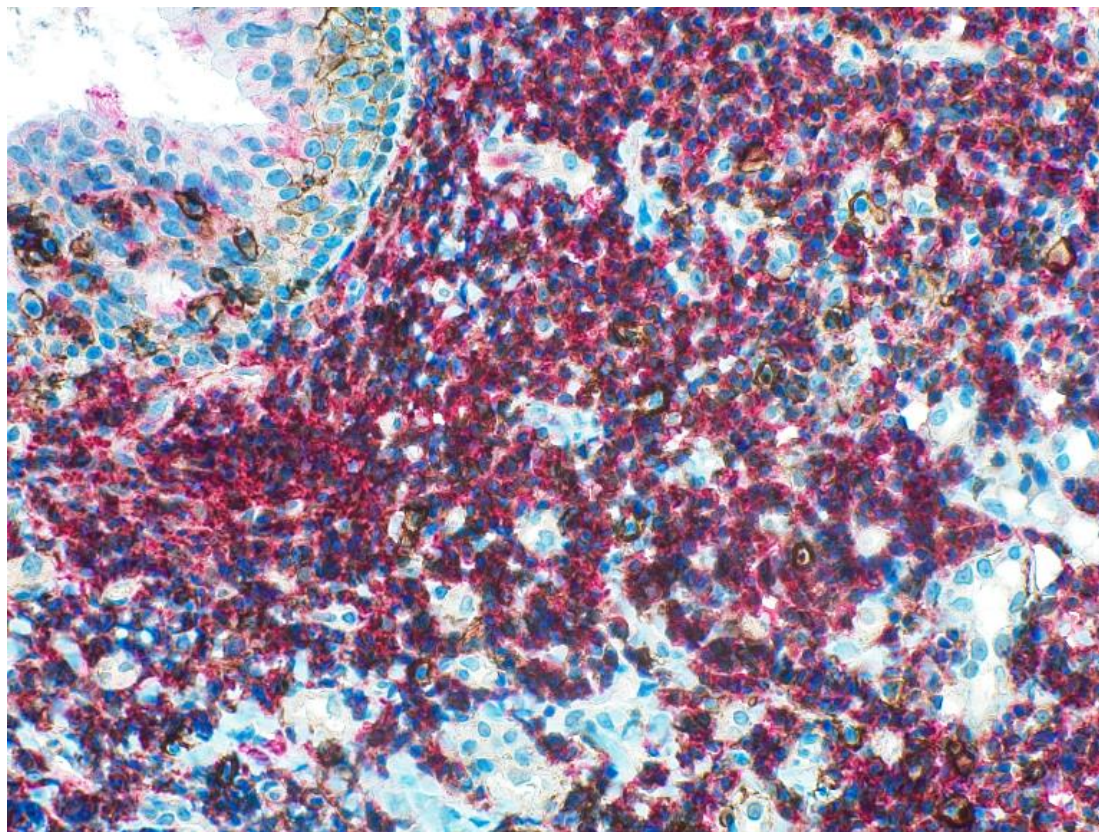

CD24 in immune cells

Supplement: Supplementary file 1 [file cancers-17-02094-s001.zip › Supplementary Figure S3.pdf]

**ALDH<sup>hi</sup> (+++)**

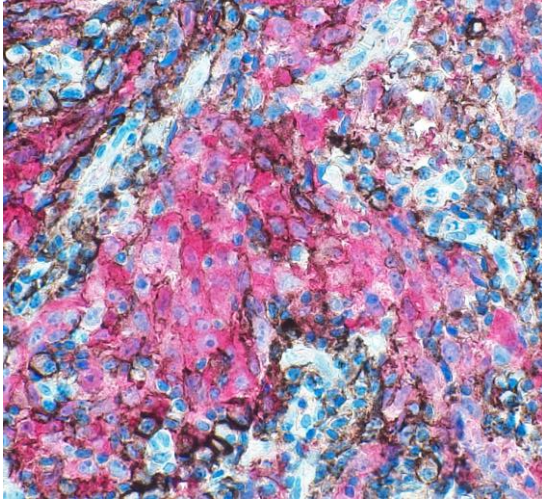

**ALDH<sup>md</sup> (++)**

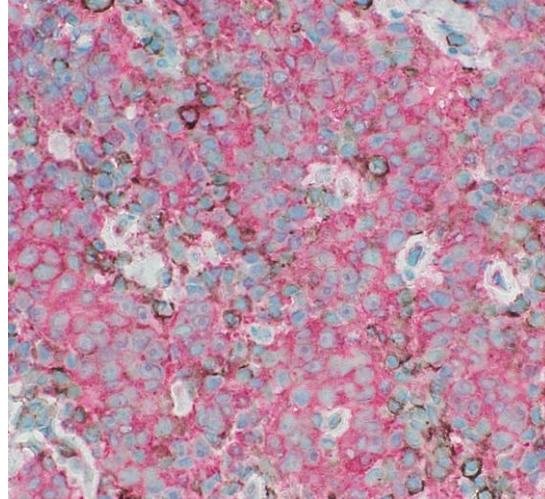

**ALDH<sup>lo</sup> (+)**

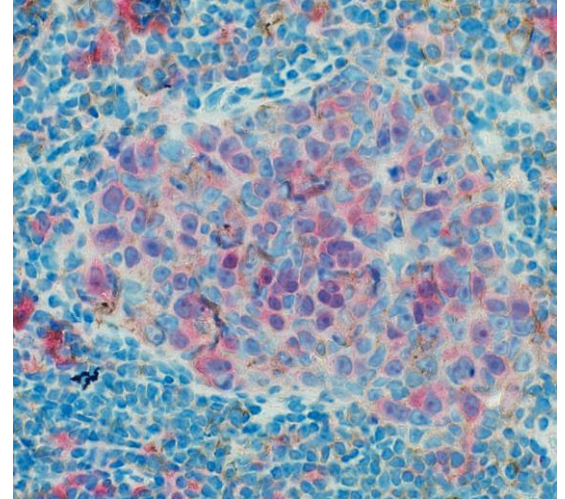

**CD24<sup>hi</sup> (+++)**

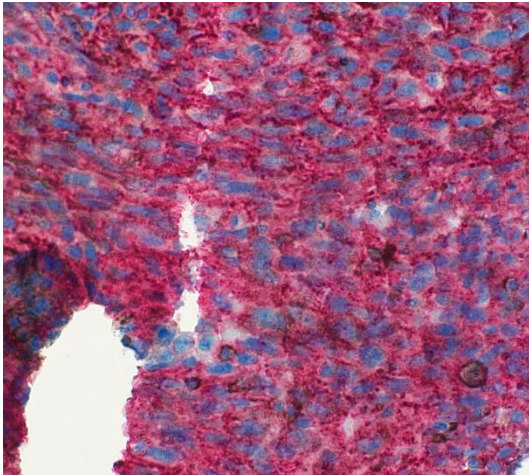

**CD24<sup>md</sup> (++)**

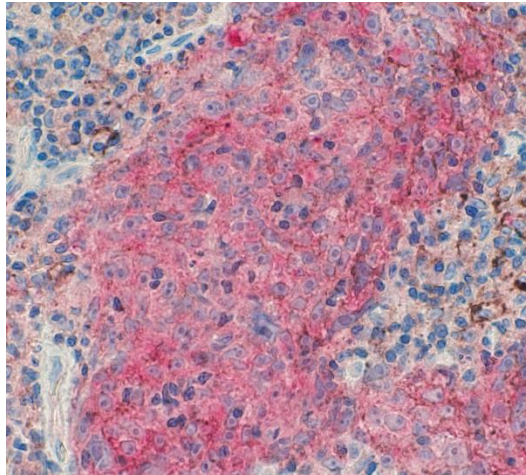

**CD24<sup>lo</sup> (+)**

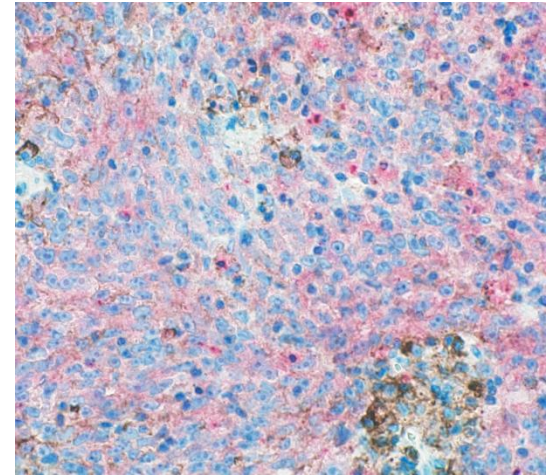

Supplement: Supplementary file 1 [file cancers-17-02094-s001.zip › Supplementary Figure S4.pdf]

**DFS**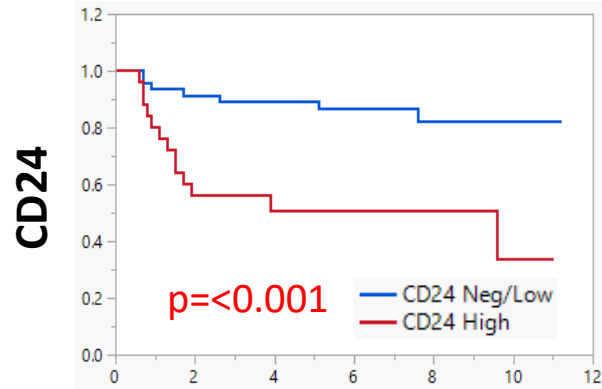**MFS**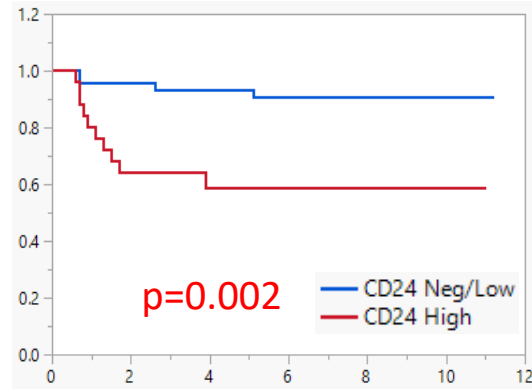**OS**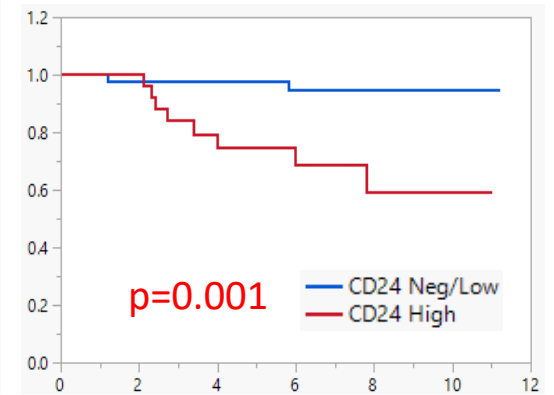**ALDH1**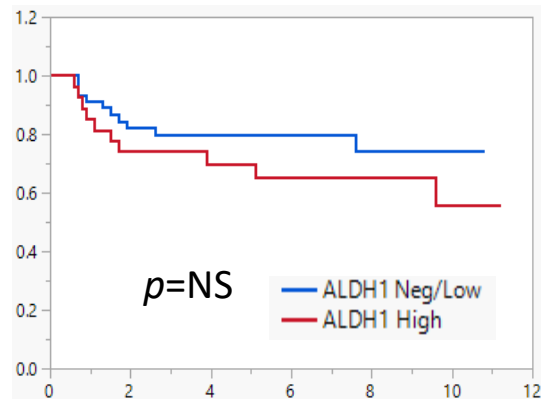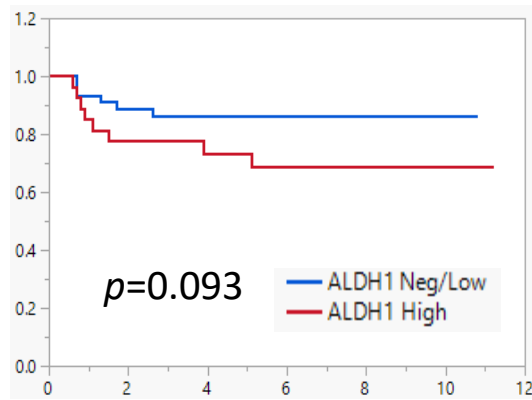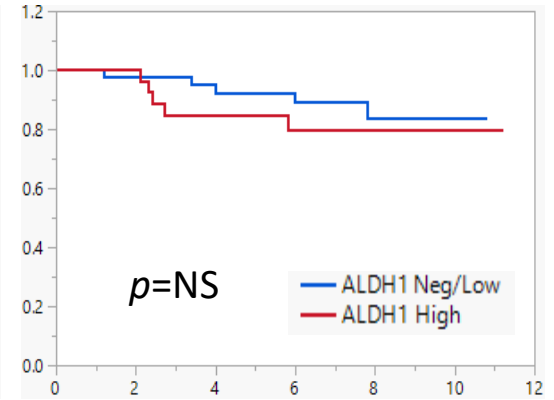

Survival (fraction)

Time to Event (Years)

Supplement: Supplementary file 1 [file cancers-17-02094-s001.zip › Supplementary Figure S5.pdf]
